# Upregulation of microRNA miR-652-3p is a prognostic risk factor for hepatocellular carcinoma and regulates cell proliferation, migration, and invasion

**DOI:** 10.1080/21655979.2021.1979861

**Published:** 2021-10-05

**Authors:** Xiaobin Chi, Yi Jiang, Yongbiao Chen, Lizhi Lv, Jianwei Chen, Fang Yang, Xiaojin Zhang, Fan Pan, Qiucheng Cai

**Affiliations:** Department of Hepatobiliary Surgery, 900 Hospital of the Joint Logistics Team, Fuzhou, China

**Keywords:** miR-652-3p, prognosis, progression, hepatocellular carcinoma

## Abstract

As powerful regulatory factors, microRNAs (miRNAs) are involved in tumor progression. The current research aimed to excavate the prognostic significance and potential regulatory mechanisms of miR-652-3p in hepatocellular carcinoma (HCC). Expression of miR-652-3p in HCC tissues and cells was exposed by Quantitative real-time polymerase chain reaction (RT-qPCR) assay, and we found that miR-652-3p was elevated in HCC tissues and cells than in the control group (*P* < 0.05). Then, the relationship between miR-652-3p levels and clinical characteristics was obtained from the Chi-square test. Kaplan-Meier survival analysis and Cox regression model to explore the outcome of miR-652-3p on the prognosis of HCC. The results investigated that overexpression of miR-652-3p was related to clinical tumor-node-metastasis (TNM) stage (*P* = 0.020) and differentiation (*P* = 0.031). HCC patients with elevated miR-652-3p levels were correlated with poor overall survival (log-rank, *P* = 0.007), and maybe a possible prognostic marker for HCC. Finally, CCK-8, colony formation, wound healing and Transwell assay was detected after transfection of HCC cells with miR-652-3p mimic or inhibitor. And the results confirmed that elevation miR-652-3p promoted the proliferation, migration, and invasion of tumor cells (*P* < 0.05). All data indicated that elevated miR-652-3p is a prognostic marker and would be able to participate in tumor progression of HCC by regulating cell proliferation, migration, and invasion.

## Introduction

Hepatocellular carcinoma (HCC) is a common type of primary liver cancer and the fifth largest malignant tumor in the world [1]. Due to its high invasiveness and mortality, this malignant tumor of the digestive system has become a major threat to human health [2,3]. Although comprehensive treatment strategies such as surgical resection, chemoembolization, liver transplantation have been successfully applied to the treatment of HCC patients. However, due to the high recurrence and metastasis rate of patients, the 5-year survival rate is still not ideal [4]. On this account, it is urgent to find new and reliable prognostic biomarkers for HCC treatment.

MicroRNAs are non-coding, highly conserved endogenous small RNA, consisting of 22-25 nucleotides [5]. Studies have proved that miRNAs are powerful regulators of biological processes such as embryogenesis, proliferation, apoptosis, and metastasis [6–8]. what’re more, dysregulated miRNAs have been persistent, including HCC. For example, miR-370, miR-3691-5p, miR-146a are dysregulated in HCC [9–11]. More importantly, a study identified circulating miRNAs related to the progression of HCC in rats, including miR-652-3p [12]. Among numerous miRNAs, miR-652-3p is located on chromosome Xp23. In tumors such as bladder cancer and ovarian cancer, its level was dysexpression [13,14]. And it serves as a prognostic marker for gastric cancer and a potential diagnostic indicator of acute decompensated heart failure [15,16]. However, the clinical and biological regulation of miR-652-3p in HCC has not been excavated.

Based on the above studies, we speculated that miR-652-3p might play a certain role in HCC. The main point of the current research was to verify the level of miR-652-3p in HCC patients and its clinical significance and continue to think about the regulatory role of miR-652-3p in the biological behavior of HCC cells. Current research suggests that miR-652-3p has potential significance as a novel prognostic marker and therapeutic target for HCC.

## Material and methods

### Clinical sample collection and evaluation

The current study was authorized by the 900 Hospital of The Joint Logistics Team and all participants signed informed consent. All tissue was processed and anonymize. The research methodology is following the standards stipulated in the Helsinki Declaration.

All HCC tissue samples (Tumor and matching adjacent non-tumor tissues) and complete clinical and pathological data were endorsed from 126 HCC patients who underwent surgical resection at the 900 Hospital of The Joint Logistics Team from June 2009 to December 2014. All patients were diagnosed as HCC by more than two pathologists before surgery, and patients with autoimmune disease and those who had received any antitumor treatment before surgery were excluded. All subjects were followed up for five years, including the time interval between surgery and death or last follow-up. In the present study, the pathological grading and staging of HCC patients were carried out according to AJCC TNM staging [17]. The patient’s gender, age, tumor size, and other clinical data and pathological characteristics were collected as shown in Table 1.

**Table 1.** Correlation between miR-652-3p expression levels and clinical features in HCC patients

Parameters	Cases No. (n = 126)	miR-625-3p expression		*P*
		Low (n = 57)	High (n = 69)	
Age (years)				0.858
<60	62	29	33	
≥60	64	28	36	
Gender				0.595
Male	65	31	34	
Female	61	26	35	
Tumor size (cm)				0.721
<5	69	30	39	
≥5	57	27	30	
Smoking status				
No	71	35	36	0.367
Yes	55	22	33	
Alcohol intake				
No	70	34	36	0.472
Yes	56	23	33	
AFP (ng/ml)				0.477
<400	66	32	34	
≥400	60	25	35	
Cirrhosis				
Yes	64	30	34	0.724
No	62	27	35	
Clinical TNM stage				0.020
I–II	58	33	25	
III–IV	68	24	44	
Differentiation				
Well and moderate	61	34	27	0.031
Poor and others	65	23	42	

AFP, alpha-fetoprotein.

### Cell culture and miRNA transfection

Human normal hepatocytes cell HL-7702 and HCC cell lines Huh-7, Hep3B, and HepG2 used in this study were all from the Chinese Academy of Sciences Cell Bank. Cells were cultured in a DMEM medium containing 10% FBS, 1% penicillin, and streptomycin in a moist incubator at 37°C, 5% CO_2_. When the cells grow to the growth phase, miR-652-3p mimic (miR10022709-1-5, Ribo Bio, Guangzhou, China), mimic NC (miR1N0000001-1-5, Ribo Bio, Guangzhou, China), miR-652-3p inhibitor (miR2171122085637-1-5, Ribo Bio, Guangzhou, China), inhibitor NC (miR2N0000001-1-5, Ribo Bio, Guangzhou, China) are transferred into the cells used Lipofectamine 2000 for in vitro cell function detection of miR-652-3p.

### RNA extraction and quantitative RT-PCR (RT-qPCR) assay

Isolation of HCC tissues and cells by Trizol to obtain the total RNA. The extracted RNA was reverse transcribed into cDNA by a reverse transcription kit. Finally, RT-qPCR reaction was carried out on ABI 7500 Fast Real-Time PCR system through the SYBR Green I Master Mix Kit. The primer sequence about the current research was as follows: U6 forward 5ʹ-CTCGCTTCGGCAGCACA-3ʹ and reverse 5ʹ-AACGCTTCACGAATTTGCGT-3ʹ; and miR-652-3p forward 5ʹ- GCCGAGAATGGCGCCACTAG −3ʹ and reverse 5ʹ- GTGCGTGTCGTGGAGTCG −3ʹ. The relative levels of miR-652-3p were estimated with the 2^−ΔΔCt^ and normalized to U6. The RT-qPCR assay was repeated 3 times independently, with 3 repetitions for each time.

### Cell proliferation assay

The cell proliferation ability was determined by the CCK-8. Cells transfected with miR-652-3p mimic or inhibitor were plated into 96-well culture plates at a density of 1.5 × 10^3^ cells/well. To ensure the accuracy of the experiment, 3 wells were repeated each time. Then a 10 μl of CCK-8 reagent (MedChem Express, China) was then supplemented cells every 24 h for the next 3 days. And then incubator for 1 h and measure the change in absorbance at 450 nm according to the specification.

### Colony formation assay

The HCC cells of transfected were disgested by trypsin and inoculated into 6-well plates at a concentration of 500 cells/ well, with 6 replicates per sample. Then cells were placed in an incubator and replaced with a fresh medium every 3 days. On the 15^th^ day, the medium in the well plates was removed and cleaned with PBS. It was then fixed in 4% paraformaldehyde for 20 min, followed by staining in 0.1% crystal violet for 20 min. Removed crystal violet and washed in running water until it was not shown purple. The cell plates were then photographed and then the unmber of clones was counted.

### Wound-healing assay

A wound-healing assay was performed to detect the migration [18]. The transfected cells were inoculated into 6-well plates, and mitomycin C was supplemented to inhibited cell growth when they reached 80%-90% fusion length. Then use a 200 μL pipette tip to draw two straight lines on the monolayer. After cleaning with serum-free DMEM, photographs were taken under a microscope. They were then placed in an incubator and incubated for 24 h before being photographed again under a microscope. Then the area of the scratch was counted by Image J software, and then it was calculated as (A_0_-A_24_)/A_0_ × 100%. Finally, the normalization of the results to the control groups. What’s more, there were 3 duplicate wells of all experimental groups, and each well was photographed for 4 regions.

### Transwell assay

Transwell was used to analyze cell migration and invasion capacity following previous studies [19]. Resuspend the cells transfected with miR-652-3p mimic or inhibitor in serum-free medium, and inoculated 200 μl of medium with the concentration of 5 × 10^4^ cells in the upper chamber of Transwell. Pre-coated the upper chamber of Transwell with 0.5 mg/ml of Matrigel (BD Bioscience, USA) in advance to resolve the invasion, and the migration of cells without Matrigel was measured. DMEM supplemented 10% FBS was added to the lower chamber. After 24 h of culture, the non-migrated and noninvasive cells in the upper chamber were wiped off. Cells in the lower chamber were fixed with 4% paraformaldehyde, stained with 0.1% crystal violet for 15 min. Microscopically, the number of randomly counted the number of cells in 5 fields under in each of three independent Transwell replicates was calculated.

### Statistical analysis

The data of the current research were analyzed by SPSS and GraphPad Prism 7.0, and the variance between tumor and adjacent tissues of HCC was compared by student’s *t*-test. The relationship between miR-652-3p levels and clinical characteristics was obtained from the Chi-square test. Kaplan-Meier survival analysis and Cox regression model were used to explore the effect of miR-652-3p on the prognosis of HCC. Results with *P* < 0.05, which are judged to be statistically significant.

## Results

Through previous studies, we speculated that miR-652-3p may have a certain clinical value in HCC and participate in the regulation of HCC progression. Subsequently, we analyzed the levels of miR-652-3p in the 126 HCC patients and the clinical prognostic value of 5-years follow-up. In vitro, miR-652-3p levels were regulated to explore its effects on cell proliferation, migration, and invasion.

### MiR-652-3p was upregulated in HCC

**Figure 1.**
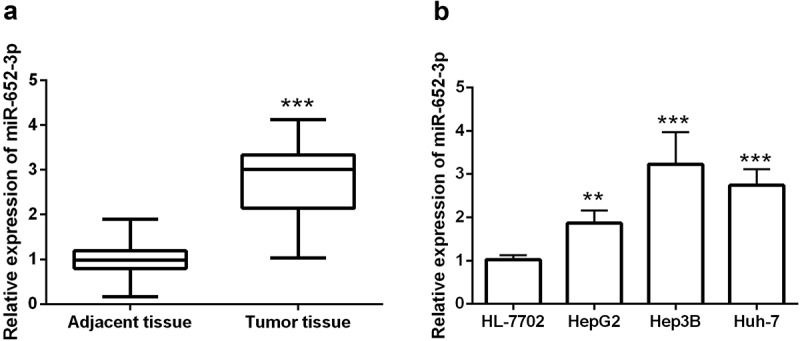
RT-qPCR assay discovered the levels of miR-652-3p in HCC tissues and cells. **a**. The levels of miR-652-3p were significantly increased in HCC tissues (***P < 0.001). **b**. Expression of miR-652-3p in HCC and human normal hepatocytes. Compared with normal cells, miR-652-3p was elevated in HCC cells (**P < 0.01)

This study detected the levels of miR-652-3p in HCC. Compared with matched adjacent tissues, miR-652-3p was remarkedly elevated in 126 HCC patients (*P*< 0.001, Figure 1(a)). We then examined the levels of miR-652-3p in HCC cells and normal hepatocytes cells HL-7702. The miR-652-3p levels in the three HCC cell lines were significantly increased (*P*< 0.01, Figure 1(b)). This is consistent with the results of the tissue samples.

### MiR-652-3p was related to clinical characteristics of HCC

Then we divided HCC patients into the high expression group of miR-652-3p (n = 69) and the low expression group of miR-652-3p (n = 57) according to the average levels (2.757 ± 0.727) of miR-652-3p in HCC patients. A Chi-square was conducted to examine the relationship between miR-652-3p and various clinicopathological characteristics of HCC, and the results were shown in Table 1. Overexpression of miR-652-3p was remarkedly correlated with clinical TNM stage (*P* = 0.020) and degree of differentiation (*P* = 0.031), but not correlated with age, gender, tumor size, AFP, Cirrhosis, smoking status, or alcohol intake (*P* > 0.05).

### Elevated miR-652-3p indicates a poor prognosis in HCC patients

**Figure 2.**
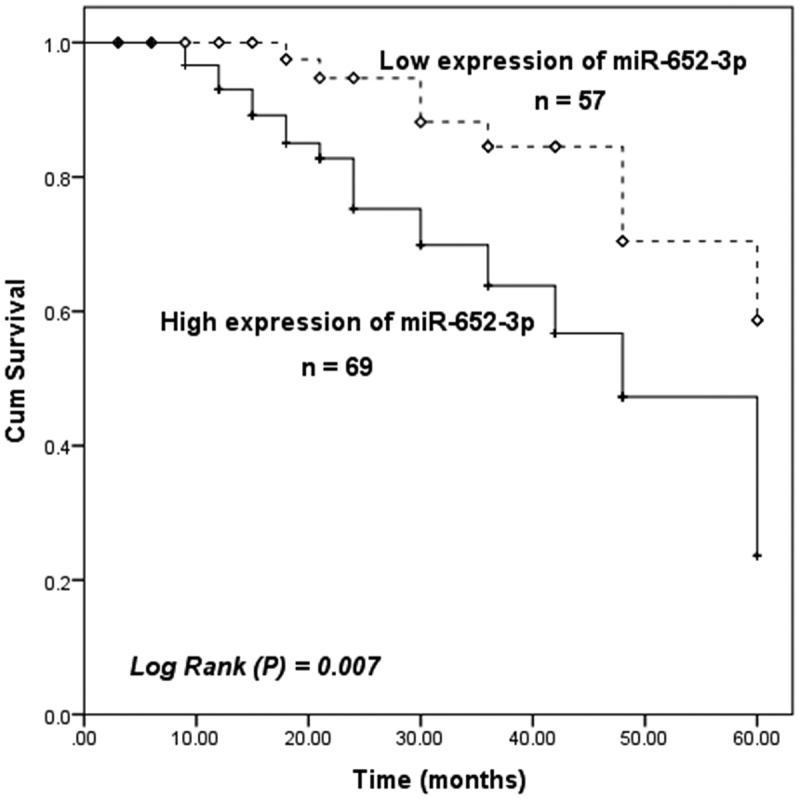
Compared with HCC patients with low miR-652-3p expression, the prognosis of patients with high miR-652-3p expression was not ideal (log-rank *P* = 0.007)

This study moreover investigated the relationship between miR-652-3p and the overall survival of HCC patients. Through the 5-year follow-up and Kaplan-Meier survival analysis. Results showed that the survival time of patients with elevated miR-652-3p levels was shorter than that of patients with low miR-652-3p levels (log-rank *P* = 0.007, Figure 2). In addition, multivariate Cox regression was performed on HCC patients and demonstrated that the upregulation of miR-652-3p was closely related to poor survival and could serve as an independent prognostic risk index for HCC patients (HR = 3.249, 95%CI = 1.323–7.979, *P* = 0.010, Table 2).

**Table 2.** Multivariate Cox analysis of miR-652-3p and clinical parameters with overall survival

Characteristics	Multivariate analysis		
	HR	95%CI	*P*
miR-1183	3.249	1.323–7.979	0.010
Age	0.442	0.308–1.673	0.443
Gender	1.086	0.445–2.595	0.853
Tumor size	0.775	0.333–1.803	0.554
Smoking status	0.647	0.299–1.399	0.268
Alcohol intake	0.697	0.317–1.533	0.370
AFP	0.560	0.238–1.317	0.184
Cirrhosis	0.406	0.168–0.981	0.045
Clinical stage	2.708	1.050–6.982	0.039
Differentiation	2.288	0.997–5.253	0.051

### MiR-652-3p regulated HCC cell function

**Figure 3.**
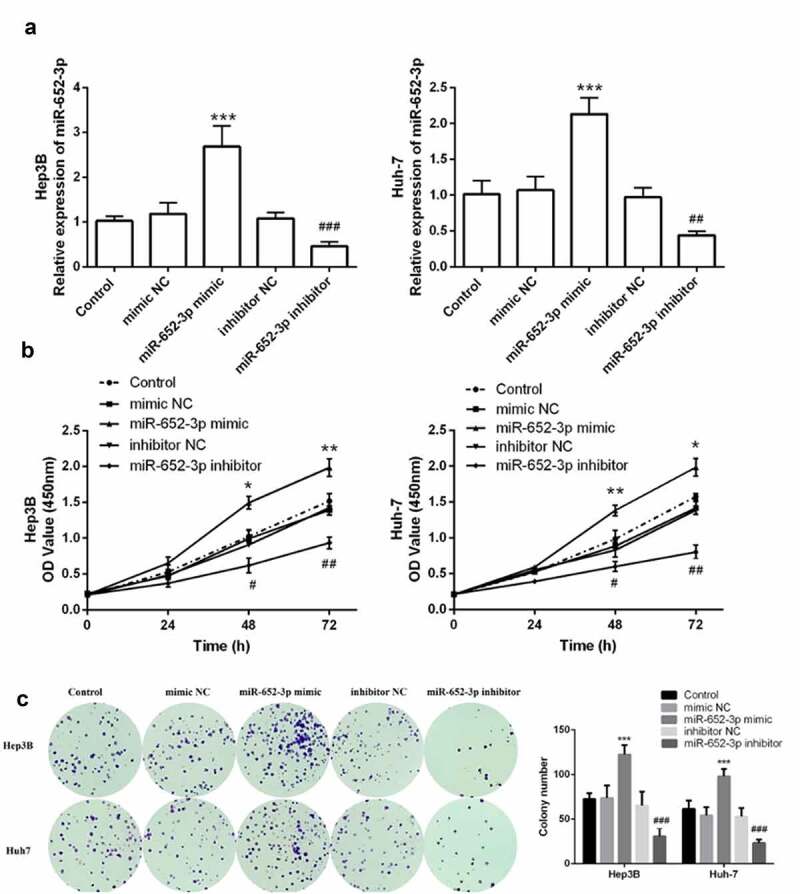
The level of miR-652-3p regulates cell proliferation of HCC cells. **a**. Expression level of miR-652-3p was detected after transfection of miR-652-3p mimics and inhibitors. (**P < 0.01). **b**. CCK-8 assay to detect the regulation of miR-652-3p on proliferation (*P < 0.05). **c**. Colony formation assay was performed to accessed the regulation of miR-652-3p on colony forming ability (***P < 0.001, compared to mimic NC; ### *P* < 0.001, compared to inhibitor NC)

Then we further explored whether miR-652-3p plays a role in the progression of HCC through in vitro cell function experiments. miR-652-3p mimic and inhibitor were transfected into HCC cells Huh-7 and Hep3B, respectively. RT-qPCR results showed that the expression of miR-652-3p in the miR-652-3p mimic group was up-regulated, and down-regulated in the miR-652-3p inhibitor group (*P* < 0.01, Figure 3(a)). The experimental outcome confirmed that the miR-652-3p level could be regulated in vitro.

**Figure 4.**
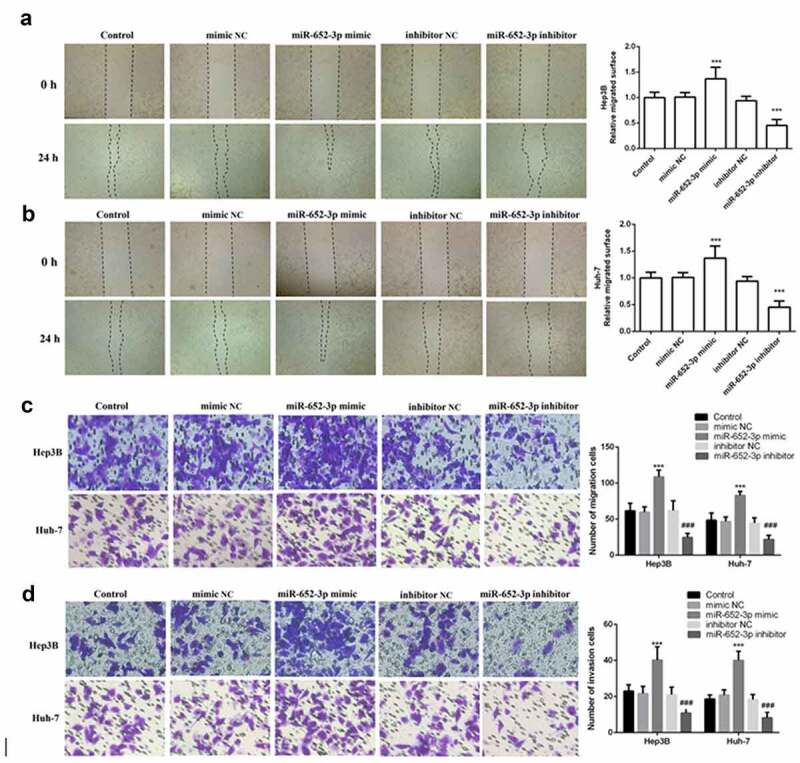
Wound healing and Transwell assay was used to detect cell migration and invasion. **a**. Wound healing assay was performed to explore the migration of Hep3B. **b**. Wound healing assay was performed to explore the migration of Huh-7. **c**. Representative images of miR-652-3p affecting cell migration and statistical results on the number of cell migration. **d**. Representative images of miR-652-3p affecting cell invasion and statistical results on the number of cell migration (***P < 0.001, compared to mimic NC; ### *P* < 0.001, compared to inhibitor NC)

Furthermore, elevated miR-652-3p significantly increased cell proliferation and colony-forming ability of Hep3B and Huh-7, while depleted miR-652-3p markedly decreased these abilities (*P* < 0.05, Figure 3(b-c)). Finally, to determine whether miR-652-3p can regulate the migration and invasion of HCC cell lines, we performed the wound healing assay and Transwell assay. In the wound healing assay, miR-652-3p mimic dramatically elevated the migration of Hep3B (Figure 4(a)) and Huh-7 (Figure 4(b)), while miR-652-3p inhibitor dramatically inhibited their migration. Similar results were observed with the Transwell assay. miR-62-3p mimic enhanced cell migration and invasion, while miR-652-3p inhibitor had the opposite effect (*P* < 0.05, Figure 4(c-d)). In conclusion, the miR-652-3p level can dramatically regulate the cell function of HCC.

## Discussion

HCC accounts for 70%-85% of primary liver cancer events. Due to its high recurrence rate and mortality, the prognosis of patients is not ideal [20,21]. Although many biomarkers areas provide the diagnosis and prognosis of HCC, such as alpha-fetoprotein (AFP), osteopontin, their clinical value is limited, and the 5-year survival rate of HCC patients is still bad, so urgently need a new and effective therapeutic prognostic biomarker or target to predict or reduce the recurrence and death of HCC [22]. Although the targeted therapy of miRNAs in clinical oncology is currently very limited, the first miRNA-targeted therapy has recently completed a phase II clinical trial. This shows that miRNA-based clinical treatment has the great potential [23].

In recent years, miRNA has been involved in the occurrence and pathogenesis of HCC, providing the possibility to discover new therapeutic targets [24–26]. For example, miR-320a-3p inhibits the invasion of HCC by targeting the regulation of c-Myc [27]. Downregulation of miR-205 can regulate the metastasis of HCC [28].

Preliminary studies have confirmed that miR-652-3p is abnormally expressed in a variety of human diseases. miR-652-3p was decreased during cerebral ischemia-reperfusion and protected rats from oxidative stress injury [29,30]. In human and rat atherosclerotic plaques, miR-652-3p is up-regulated and participates in its progression [31]. Osteosarcoma tissue and cell line miR-652-3p are significantly elevated and are involved in cell proliferation and migration [32]. miR-652-3p was remarkedly elevated in tumor tissue of patients with uveal melanoma [33]. It is worth noting that Sukata et al. found several abnormal circulating serum miRNAs closely associated with the progression of HCC in rats in a 2011 study, among the up-regulated miRNAs including miR-652-3p [12], although they later discovered differences in miRNA levels in tissues and serum. However, we first detected the levels of miR-652-3p in the tissues of 126 HCC patients and HCC cell lines. The outcome showed that miR-652-3p was elevated in HCC tissues and cell lines. To verify these results, we verified the online databases ENCORI and a large-scale miRnome screen (Supplementary Figure). The experimental results confirmed that miR-652-3p was significantly increased in HCC, which is consistent with our results.

Analyzing miR-652-3p in HCC patients and its relationship with clinicopathological characteristics, it is found that the overexpression of miR-652-3p was significantly related to the clinical TNM stage and differentiation of HCC patients. Therefore, we believe that miR-652-3p may be the oncogene of HCC, delaying the progression of this malignant tumor. Furthermore, we also found that patients with elevated miR-652-3p have a lower survival rate. And the experimental results suggested that the increase of miR-652-3p expression level was correlated with poor survival. At the same time, the consequence of the multivariate Cox model demonstrated that the elevated miR-652-3p can be used as an independent prognostic risk indicator for HCC.

What’s more, through cell function experiments, we studied the role of miR-652-3p in the progression of HCC. The outcome showed that overexpression of miR-652-3p could significantly promote cell proliferation, migration, and invasion. The results confirmed that the elevated miR-652-3p could promote the tumor progression of HCC. miRNA plays its specific function by regulating its target genes, so we try to explore them. Previous studies have found that Kuppel-like factor 9 (KLF9) is a potential target gene of miR-652 and participated in the regulation of proliferation and invasion of osteosarcoma by miR-652 [32]. And the role of KLF9 in HCC has been revealed to some extent in previous studies. KLF9 participated in the thyroid hormone triiodothyronine (T3) to inhibit the development of HCC [34]. Moreover, the upregulation of KLF9 inhibits the proliferation and migration of HCC cells and induces apoptosis [35], and its ectopic expression delays the onset of HCC tumors and promotes the regression of established tumors in vivo [36]. Therefore, based on the above report, we have reason to suspect that miR-652-3p may affect the progress of HCC by inhibiting KFL9. However, this is only our conjecture and further research and verification are needed. Although studies on the role of miRNA in the progression of HCC have been reported and confirmed many times, for example, miR-125b-2-3p [37], miR-590-3p [38], and miR-139-5p [39]. However, to our knowledge, this is the first time to confirm that expression pattern of miR-652-3p in HCC, and the high level is associated with clinical progress and may be involved in the progression of HCC by regulating the proliferation, migration, and invasion of HCC cells.

This study has certain limitations. Due to the small samples size, there was no significant correlation between the expression level of miR-652-3p and AFP. Therefore, it is necessary to expand the sample size for further studies. What’s more, fatty liver is one of the risk factors of HCC, and changes in HCC phenotypes play a crucial role in its progression, However, this study failed to timely explore the important role of fatty liver and miR-652-3p in HCC, which is also the main limitation of this study. This will be taken into account in our further research.

## Conclusion

To make a long story short, we confirmed that miR-652-3p is an oncogene in HCC, which may promote the progression of HCC by promoting cell proliferation, migration, and invasion. Moreover, miR-652-3p can save as a potential prognostic marker.

## Supplementary Material

Supplemental Material

## Data Availability

The corresponding author can provide the data of this study if reasonable.

## Research highlights

- miR-652-3p was upregulated in both HCC tissues and cell lines.
- Elevated miR-652-3p is a good indicator of poor prognosis in HCC patients.
- miR-652-3p promoted the proliferation, migration, and invasion of HCC cell lines.

## References

[1] Ferlay J, Shin HR, Bray F, et al. Estimates of worldwide burden of cancer in 2008: GLOBOCAN 2008. Int J Cancer. 2010;127(12):2893–2917. 21351269 10.1002/ijc.25516

[2] Siegel RL, Miller KD, Jemal A. Cancer statistics, 2018. CA Cancer J Clin. 2018;68(1):7–30. 29313949 10.3322/caac.21442

[3] Venook AP, Papandreou C, Furuse J, et al. The incidence and epidemiology of hepatocellular carcinoma: a global and regional perspective. Oncologist. 2010;15(Suppl 4):5–13. 10.1634/theoncologist.2010-S4-05 21115576

[4] Hu X, Wang R, Ren Z, et al. MiR-26b suppresses hepatocellular carcinoma development by negatively regulating ZNRD1 and Wnt/β-catenin signaling. Cancer Med. 2019;8(17):7359–7371. 10.1002/cam4.2613 PMC6885897 31637871

[5] Stark A, Brennecke J, Russell RB, et al. Identification of drosophila MicroRNA targets. PLoS Biol. 2003;1(3):E60. 14691535 10.1371/journal.pbio.0000060 PMC270017

[6] Bartel DP. MicroRNAs: genomics, biogenesis, mechanism, and function. Cell. 2004;116(2):281–297. 14744438 10.1016/s0092-8674(04)00045-5

[7] Jin J, Zhang J, Xue Y, et al. miRNA-15a regulates the proliferation and apoptosis of papillary thyroid carcinoma via regulating AKT pathway. Onco Targets Ther. 2019;12:6217–6226. 31496725 10.2147/OTT.S213210 PMC6689766

[8] Peng P, Chen T, Wang Q, et al. Decreased miR-218-5p levels as a serum biomarker in bone metastasis of prostate cancer. Oncol Res Treat. 2019;42(4):165–185. 30870834 10.1159/000495473

[9] Liu Z, Ma M, Yan L, et al. miR-370 regulates ISG15 expression and influences IFN-α sensitivity in hepatocellular carcinoma cells. Cancer Biomarkers. 2018;22(3):453–466. 29758929 10.3233/CBM-171075 PMC6027951

[10] Du W, Zhang X, Wan Z. miR-3691-5p promotes hepatocellular carcinoma cell migration and invasion through activating PI3K/Akt signaling by targeting PTEN. Onco Targets Ther. 2019;12:4897–4906. 31417285 10.2147/OTT.S208127 PMC6593750

[11] Wang H, Li X, Li T, et al. Multiple roles of microRNA-146a in immune responses and hepatocellular carcinoma. Oncol Lett. 2019;18(5):5033–5042. 31612014 10.3892/ol.2019.10862 PMC6781720

[12] Sukata T, Sumida K, Kushida M, et al. Circulating microRNAs, possible indicators of progress of rat hepatocarcinogenesis from early stages. Toxicol Lett. 2011;200(1–2):46–52. 21035526 10.1016/j.toxlet.2010.10.013

[13] Zhu QL, Zhan DM, Chong YK, et al. MiR-652-3p promotes bladder cancer migration and invasion by targeting KCNN3. Eur Rev Med Pharmacol Sci. 2019;23(20):8806–8812. 31696467 10.26355/eurrev_201910_19275

[14] Zhao Y, Hu Y, Shen Q, et al. CircRNA_MYLK promotes malignant progression of ovarian cancer through regulating microRNA-652. Eur Rev Med Pharmacol Sci. 2020;24(10):5281–5291. 32495861 10.26355/eurrev_202005_21310

[15] Li J, Zou X. MiR-652 serves as a prognostic biomarker in gastric cancer and promotes tumor proliferation, migration, and invasion via targeting RORA. Cancer Biomarkers. 2019;26(3):323–331. 31524147 10.3233/CBM-190361 PMC12826413

[16] Liu J, Zhang H, Li X, et al. Diagnostic and prognostic significance of aberrant miR-652-3p levels in patients with acute decompensated heart failure and acute kidney injury. J Int Med Res. 2020;48(11):300060520967829. 33249927 10.1177/0300060520967829 PMC7708706

[17] Edge SB, Compton CC. The American Joint Committee on Cancer: the 7th edition of the AJCC cancer staging manual and the future of TNM. Ann Surg Oncol. 2010;17(6):1471–1474. 20180029 10.1245/s10434-010-0985-4

[18] Chen L, Zhu Q, Lu L, et al. MiR-132 inhibits migration and invasion and increases chemosensitivity of cisplatin-resistant oral squamous cell carcinoma cells via targeting TGF-β1. Bioengineered. 2020;11(1):91–102. 31906769 10.1080/21655979.2019.1710925 PMC6961592

[19] Gao F, Wu H, Wang R, et al. MicroRNA-485-5p suppresses the proliferation, migration and invasion of small cell lung cancer cells by targeting flotillin-2. Bioengineered. 2019;10(1):1–12. 30836864 10.1080/21655979.2019.1586056 PMC6527069

[20] Torre LA, Bray F, Siegel RL, et al. Global cancer statistics, 2012. CA Cancer J Clin. 2015;65(2):87–108. 25651787 10.3322/caac.21262

[21] Perz JF, Armstrong GL, Farrington LA, et al. The contributions of hepatitis B virus and hepatitis C virus infections to cirrhosis and primary liver cancer worldwide. J Hepatol. 2006;45(4):529–538. 16879891 10.1016/j.jhep.2006.05.013

[22] Zhang Z, Lei B, Chai W, et al. Increased expression of insulin-like growth factor-1 receptor predicts poor prognosis in patients with hepatocellular carcinoma. Medicine (Baltimore). 2019;98(44):e17680. 31689787 10.1097/MD.0000000000017680 PMC6946458

[23] Janssen HL, Reesink HW, Lawitz EJ, et al. Treatment of HCV infection by targeting microRNA. N Engl J Med. 2013;368(18):1685–1694. 23534542 10.1056/NEJMoa1209026

[24] Li J, Jin B, Wang T, et al. Serum microRNA expression profiling identifies serum biomarkers for HCV-related hepatocellular carcinoma. Cancer Biomarkers. 2019;26(4):501–512. 10.3233/CBM-181970 PMC12826393 31658041

[25] Ding W, Yang H, Gong S, et al. Candidate miRNAs and pathogenesis investigation for hepatocellular carcinoma based on bioinformatics analysis. Oncol Lett. 2017;13(5):3409–3414. 28521446 10.3892/ol.2017.5913 PMC5431310

[26] Otsuka M, Kishikawa T, Yoshikawa T, et al. MicroRNAs and liver disease. J Hum Genet. 2017;62(1):75–80. 27225852 10.1038/jhg.2016.53

[27] Ye Y, Song Y, Zhuang J, et al. MicroRNA-302a-3p suppresses hepatocellular carcinoma progression by inhibiting proliferation and invasion. Onco Targets Ther. 2018;11:8175–8184. 30573969 10.2147/OTT.S167162 PMC6292402

[28] Lu J, Lin Y, Li F, et al. MiR-205 suppresses tumor growth, invasion, and epithelial-mesenchymal transition by targeting SEMA4C in hepatocellular carcinoma. FASEB J. 2018;fj201800113R. 10.1096/fj.201800113R 29799789

[29] Zuo ML, Wang AP, Song GL, et al. miR-652 protects rats from cerebral ischemia/reperfusion oxidative stress injury by directly targeting NOX2. Biomed Pharmacother. 2020;124:109860. 32000043 10.1016/j.biopha.2020.109860

[30] Gao JD, Li RJ, Ma PL, et al. Knockdown of lncRNA HCP5 protects against cerebral ischemia/reperfusion injury by regulating miR-652-3p. J Biol Regul Homeost Agents. 2020;34(3):893–900. 32657103 10.23812/20-148-A-33

[31] Huang R, Hu Z, Cao Y, et al. MiR-652-3p inhibition enhances endothelial repair and reduces atherosclerosis by promoting Cyclin D2 expression. EBioMedicine. 2019;40:685–694. 30674440 10.1016/j.ebiom.2019.01.032 PMC6413686

[32] Jin Y, Yang L, Li X. MicroRNA-652 promotes cell proliferation and osteosarcoma invasion by directly targeting KLF9. Exp Ther Med. 2020;20(4):2953–2960. 32855660 10.3892/etm.2020.9037 PMC7444349

[33] Xia Z, Yang C, Yang X, et al. miR-652 promotes proliferation and migration of Uveal Melanoma cells by targeting HOXA9. Med Sci Monit. 2019;25:8722–8732. 31740654 10.12659/MSM.917099 PMC6880646

[34] Kowalik MA, Puliga E, Cabras L, et al. Thyroid hormone inhibits hepatocellular carcinoma progression via induction of differentiation and metabolic reprogramming. J Hepatol. 2020;72(6):1159–1169. 31954205 10.1016/j.jhep.2019.12.018

[35] Fu DZ, Cheng Y, He H, et al. The fate of Kruppel-like factor 9-positive hepatic carcinoma cells may be determined by the programmed cell death protein 5. Int J Oncol. 2014;44(1):153–160. 24173774 10.3892/ijo.2013.2147

[36] Sun J, Wang B, Liu Y, et al. Transcription factor KLF9 suppresses the growth of hepatocellular carcinoma cells in vivo and positively regulates p53 expression. Cancer Lett. 2014;355(1):25–33. 25242357 10.1016/j.canlet.2014.09.022

[37] Huang HQ, Chen G, Xiong DD, et al. Down-regulation of microRNA-125b-2-3p is a risk factor for a poor prognosis in hepatocellular carcinoma. Bioengineered. 2021;12(1):1627–1641. 33949293 10.1080/21655979.2021.1921549 PMC8806266

[38] Zhang H, Liu S, Chen L, et al. MicroRNA miR-509-3p inhibit metastasis and epithelial-mesenchymal transition in hepatocellular carcinoma. Bioengineered. 2021;12(1):2263–2273. 34115554 10.1080/21655979.2021.1932210 PMC8806452

[39] Zhu X, Jiang S, Wu Z, et al. Long non-coding RNA TTN antisense RNA 1 facilitates hepatocellular carcinoma progression via regulating miR-139-5p/SPOCK1 axis. Bioengineered. 2021;12(1):578–588. 33517826 10.1080/21655979.2021.1882133 PMC8291788

