# Supplementary material for: Upregulation of microRNA miR-652-3p is a prognostic risk factor for hepatocellular carcinoma and regulates cell proliferation, migration, and invasion
Source: Bioengineered. 2021 Oct 5;12(1):7519–28. doi: 10.1080/21655979.2021.1979861 (PMC8806865; doi:10.1080/21655979.2021.1979861)
Supplement: Supplemental Material [file KBIE_A_1979861_SM0036.zip › supplementary/Supplementary Figure legend.docx]

**Supplementary Figure** Online databases ENCORI and miRnome screen both confirmed that miR-652-3p was upregulated in HCC tissues (LIHC was the liver hepatocellular carcinoma).
